# miR-155 induction in microglial cells suppresses Japanese encephalitis virus replication and negatively modulates innate immune responses

**DOI:** 10.1186/1742-2094-11-97

**Published:** 2014-05-29

**Authors:** Siddhika Pareek, Saugata Roy, Bharti Kumari, Pratistha Jain, Arup Banerjee, Sudhanshu Vrati

**Affiliations:** 1 Vaccine and Infectious Disease Research Center, Translational Health Science and Technology Institute, 496, Udyog Vihar Phase-III, Gurgaon 122016, India

**Keywords:** NeurimmiRs, JEV, *CD45*, Microglia activation

## Abstract

**Background:**

Microglial cells, which are resident macrophages of the central nervous system, play important roles in immune responses and pathogenesis. Japanese encephalitis virus (JEV) is a neurotropic virus that infects microglial cells in brain. Several microRNAs including miR-155 and miR-146a play an important role in defining the microglia inflammatory profile. In this study, we have investigated the effect of miR-155 and miR-146a modulation on JEV infection as well as innate immune responses in human microglial cells.

**Methods:**

*In vitro* studies were performed in JEV-infected human microglial CHME3 cells. miR-155 or miR-146a were overexpressed and total RNA and protein were extracted following JEV-infection. Expression of genes involved in innate immune responses was studied by PCR array, quantitative real-time PCR (qPCR), western blot and Fluorescence activated cell sorter (FACS). JEV replication was monitored by studying the viral RNA by qPCR, protein by western blot, and titres by plaque assay.

**Results:**

Overexpression of miR-155 in CHME3 cells resulted in significantly reduced JEV replication whereas miR-146a overexpression had an insignificant effect. Additionally, interferon regulatory factor 8 (*IRF8*) and complement factor H (*CFH*) were induced during JEV infection; however, this induction was attenuated in miR-155 overexpressing cells following JEV infection. Further, JEV-induced *NF-κB* regulated downstream gene expression was attenuated. Interestingly, an increased level of *CD45*, a negative regulator of microglia activation and a reduced phosphorylated-Signal Transducers and Activators of Transcription (*p-STAT1*) expression was observed in miR-155 overexpressing cells upon JEV infection.

**Conclusion:**

Induction of miR-155 in human microglial cells may negatively modulate JEV-induced innate immune gene expression and may have a beneficial role in limiting JEV replication in human microglial cells.

## Background

Japanese encephalitis (JE) is an acute central nervous system (CNS) inflammatory disease caused by infection with Japanese encephalitis virus (JEV); a small, enveloped, plus-strand RNA virus belonging to the *Flaviviridae* family. It is the leading cause of viral encephalitis in south-east Asia, India, and China where three billion people are at risk of contracting the disease, yet its pathogenesis remains poorly understood. While neurons are believed to be the primary target of JEV in the brain, a recent report has suggested that microglial cells can be directly infected with JEV [1,2]. Microglial cells are the resident immune cells of the CNS and have a critical role in host defense against invading pathogens. However, substantial evidence suggests that infection of microglia by JEV may actually lead to neuronal cell death through uncontrolled production of pro-inflammatory cytokines. Therefore, downregulation of cytokine production can serve to dampen the inflammatory response and can contribute to better virus clearance and increased protection against JEV [3]. Indeed, inhibition of chronic neuroinflammation, particularly due to microglial activation, has been suggested to be a practical strategy in the treatment of neurodegenerative diseases [4,5].

Recently, a new class of regulatory RNAs, called microRNAs (miRNAs) have emerged that modulate immune response and play key regulatory roles in virus-host interactions. These miRNAs serve as universal regulators of differentiation, activation, and polarization of mammalian cells including microglia and macrophages in normal and diseased CNS [6]. Thus, modulation of cellular miRNA expression during viral infection may be an important determinant of disease outcome [7]. Recent reports suggested that a subset of miRNAs, termed as NeurimmiRs, co-exist in the brain and peripheral organs [8,9]. These miRNAs can affect both neuronal and immune functions and thus constitute important therapeutic targets for those diseases that affect both the immune system and brain functions. Among them, miR-155 and miR-146a are multifunctional and widely reported to modulate different stages of innate immune response during inflammation and infection [10-12]. Thus, miR-155 and miR-146a were shown to increase in the microglial cells in response to stimulation with Lipopolysaccharides (LPS) and Polyinosinic-polycytidylic acid (poly(I:C), respectively, and they seem to play a fundamental role in the microglial inflammatory profile [13,14]. These miRNAs are also associated with interferon (IFN) signaling pathways [15,16]. Moreover, miR-155 and miR-146a not only modulate Toll-like receptors (TLRs)-mediated innate immune response, but also target complement regulatory proteins and facilitate complement activation [17-19]. This phenomenon is very important to eliminate the virus from infected cells. Furthermore, both miR-146a and miR-155 have been shown to play an important role in viral infection. For example, Wu *et al.*[20] reported an increased dengue virus 2 (DENV2) replication in miR-146a overexpressing cells, whereas overexpression of miR-155 significantly suppressed human immunodeficiency virus (HIV) infection in activated macrophages [21].

Since JEV is a neurotropic virus it is likely that NeurimmiRs play an important role in virus replication and immunopathology. Using a global miRNA array we have identified differentially expressed NeurimmiRs in human microglial cells during the course of JEV infection. Of these, we have focused our study on miR-155 and miR-146a and have investigated their effect on JEV replication and their role in the modulation of microglia-mediated innate immune response during JEV infection. For this purpose, *in vitro* studies were performed in JEV-infected human microglial CHME3 cells. Our results indicate that miR-155 induction might have a beneficial role for the host by limiting JEV replication through modulation of microglia-mediated innate immune responses.

## Materials and methods

### Cells, antibodies, miRNA mimics, and inhibitors

Human microglial cells (CHME3) were provided by the National Brain Research Centre, Manesar, India. Porcine stable kidney (PS) cell line was procured from National Centre for Cell Science, Pune, India. CHME3 cells were grown in Dulbecco’s Modified Eagle’s Medium (DMEM, Invitrogen, Carlsbad, CA, USA) and PS cells in Eagle’s Minimal Essential Medium (MEM, Invitrogen, Carlsbad, CA, USA) supplemented with 10% fetal bovine serum (FBS), 2 mM L-glutamine, and 100 μg/ml penicillin-streptomycin (Invitrogen, Carlsbad, CA, USA). Primary antibodies against *MyD88, Ikkϵ, IRF8, p-STAT1, STAT1* and HRP-conjugated secondary antibodies were purchased from Cell Signaling Technology, Beverly, MA, USA. *CD-45* antibodies were from BD Biosciences, San jose, CA, USA. Rabbit antibody against JEV *NS1* protein was produced in-house. MicroRNA mimics and inhibitors were from Invitrogen, Carlsbad, CA, USA (Assay ID: MC12601, mimic for hsa-miR-155-5p; MH12601, inhibitor for hsa-miR-155-5p; MC10722, mimic for hsa-miR-146a-5p; MH10722, inhibitor for hsa-miR-146a-5p, *mir*Vana® miRNA Mimic Control #1). NF-κB inhibitor ammonium pyrrolidine dithiocarbamate, and PI3K inhibitor LY294002 were from Sigma-Aldrich, Saint Louis, MO, USA.

### Transfection of cells and virus infection

The P20778 strain of JEV was propagated in PS cells and titrated by plaque assay [22]. Microglial cells were seeded in 6-well tissue culture plates at a density of 0.5 × 10^6^ cells/well and transfected 24 hours later using Lipofectamine 2000® reagent (Invitrogen, Carlsbad, CA, USA) according to the manufacturer’s protocol. For overexpression or inhibition studies, 25 pmol of a miRNA mimic or inhibitor, respectively, was transfected. Cells were washed with 1 × PBS after 24 hours transfection and infected with JEV at multiplicity of infection 5 (MOI = 5). Culture supernatant was collected for virus titration as plaque forming unit (PFU)/ml and cells lysate was used for protein and RNA studies at different times post-infection (pi).

### RT^2^ Profiler PCR array

The Human NFκB Signaling Targets PCR array (#PAHS-0225Z) (SA Biosciences/Qiagen, Hilden, Germany) was used to determine the profile of genes associated with the human innate and adaptive immune responses. Total RNA was extracted from JEV-infected and uninfected or specific mimic- and inhibitor-transfected CHME3 cells using RNeasy mini kit (Qiagen, Hilden, Germany) with inclusion of a DNase I treatment step. cDNA was prepared from 1 μg total RNA using a RT^2^ PCR array first strand kit (Qiagen, Hilden, Germany). Quantitative real-time PCR (qPCR) was performed with an ABI PRISM 7500 (Applied Biosystems, Foster City, CA, USA) according to the manufacturer’s instructions.

### Western blot analysis

Control and treated microglial cells were washed with 1 × PBS and lysed in cell lysis buffer (Sigma-Aldrich, Saint Louis, MO, USA) in the presence of protease inhibitor cocktail (Roche Diagnostics, Basel, Switzerland). The concentration of the protein lysate was determined using the Bradford method. The protein sample (50 μg) was electrophoresed on 10% sodium dodecyl sulfate-polyacrylamide gel (SDS-PAGE) and transferred onto a nitrocellulose membrane. The membrane was then blocked using 5% non-fat dry milk in PBST (PBS containing 0.05% Tween-20) for one hour at room temperature on a shaker. After blocking, the membrane was incubated with rabbit anti-human primary antibody overnight at 4°C with gentle shaking. After three washes of 10 minutes each with 1 × PBST, blots were incubated with anti-rabbit horseradish peroxidase (HRP) conjugated secondary antibody for one hour with gentle shaking at room temperature. After three washes of the membrane for 10 minutes each with PBST the western blots were developed using chemiluminescence reagents (Santa Cruz Biotechnology, Dallas, Texas, USA). Afterwards membranes were stripped using stripping buffer (10% SDS, Tris-Cl pH-6.8, 0.8% β-mercaptoethanol) and re-probed with Glyceraldehyde 3-phosphate dehydrogenase (GAPDH) antibodies. The protein levels were normalized with the GAPDH levels.

### Flow cytometry assay

Expression of CD-45 isoforms on CHME3 cells was examined by fluorescence activated cell sorter (FACS) analysis. Cells were fixed using 2% para-formaldehyde and stained with CD-45 antibody conjugated with Alexa-488 dye (BD Biosciences, San jose, CA, USA) as per the manufacturer’s instruction. Samples were analyzed on FACS Calibur cell analyser (BD Biosciences, San jose, CA, USA). Data were analyzed using FlowJo flow cytometry analysis software (Tree Star Inc, Ashland, OR, USA).

### Quantitation of mRNA expression

Cells were lysed in TRIzol reagent (Invitrogen, Carlsbad, CA, USA) for RNA isolation and the total RNA was isolated using RNeasy kit (Qiagen, Hilden, Germany). RNA concentration was quantified using a NanoDrop 2000 spectrophotometer (Thermo Fisher, Waltham, MA, USA). RNA (1 μg) was reverse transcribed using prime script first-strand cDNA Synthesis kit (Takara Bio Inc. Otsu, Shiga, Japan) in a 20 μl reaction according to the manufacturer’s protocol. The expression of cellular genes was studied by qPCR with the fluorescent DNA-binding dye SYBR green (Power SYBR Green PCR master kit; Applied Biosystems, Foster City, CA, USA) by the real-time fluorescence detection method. JEV RNA was quantified as described earlier [23]. Each quantitative PCR reaction was performed in triplicate and the mean threshold cycle (Ct) value for each sample was used for data analysis. The RNA transcript levels were normalized to that of GAPDH.

### Quantitation of miRNA expression

For detection of mature miRNAs, 10 ng total RNA was reverse transcribed *in vitro* to cDNA using the TaqMan MicroRNA Reverse Transcription kit (Applied Biosystems, Foster City, CA, USA) according to the manufacturer’s instructions. All miRNAs were assayed individually by TaqMan probe-directed real-time PCR (Reporter-FAM, Quencher-NFQ-MGB, Applied Biosystems, Foster City, CA, USA) using ABI 7500 Fast Real-Time PCR system (Applied Biosystems, Foster City, CA, USA). The following thermal cycling profile was used for the PCR analysis: 95°C for 15 minutes, 40 cycles at 94°C for 15 seconds, and 55°C for 30 seconds. Each qPCR reaction was performed in triplicate and the mean Ct value for each sample was used for data analysis. Expression levels of miRNAs were normalized to that of U6 snRNA.

### Statistical analysis

All experiments were performed in triplicate. Gene expression profiling data were analyzed statistically using one-way analysis of variance (ANOVA) following Bonferroni’s multiple comparison tests. Data were presented as the mean ± SD; statistical significance of difference (*P* value) for two means was assessed using an unpaired Student’s *t*-test using the GraphPad Prism 5 software (GraphPad, San Diego, CA, USA), and *P* < 0.05 was considered significant.

## Results

### JEV infection modulates expression of NeurimmiRs in microglial cells

**Figure 1:**
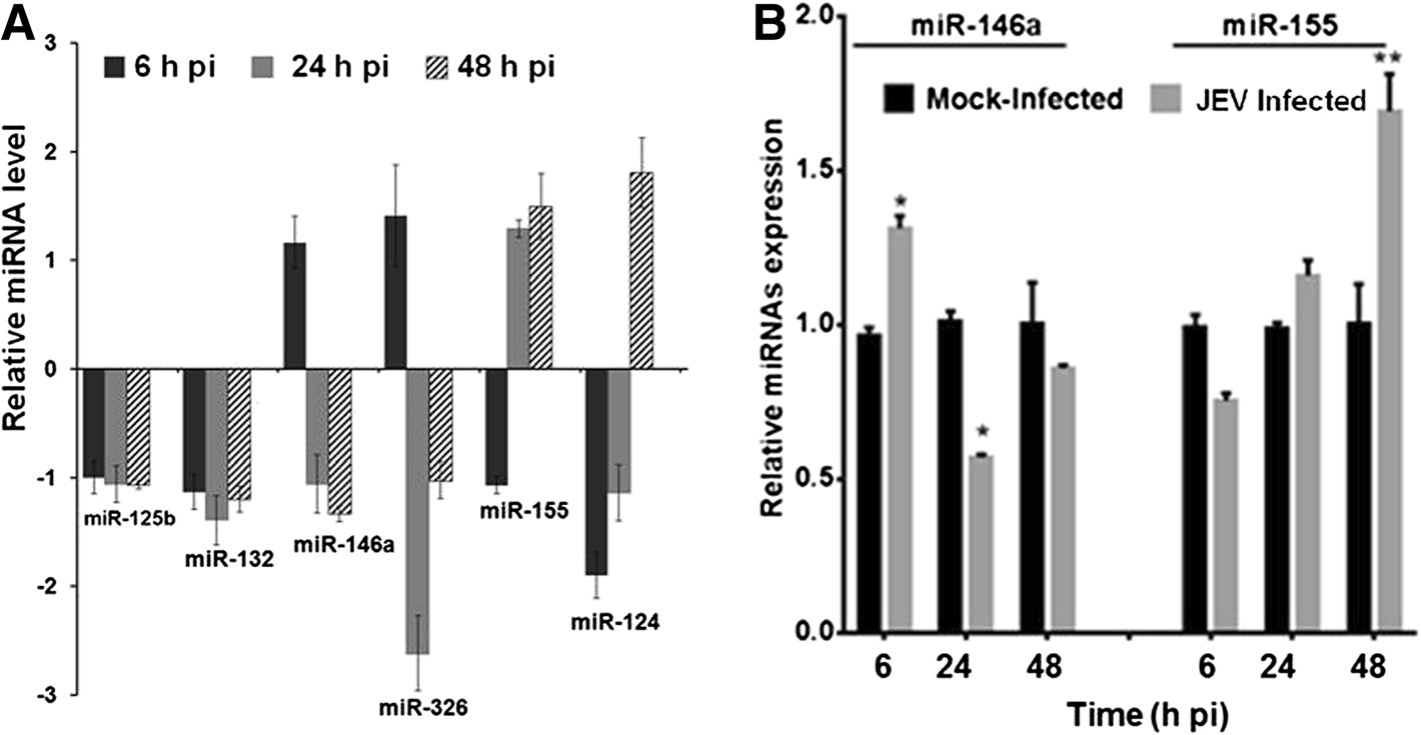
**Differential expression of NeurimmiRs in human microglial cells after JEV infection.** CHME3 cells were mock-infected or infected with JEV. Total RNA was isolated from cells at different time points. **(A)** Relative abundance of six NeurimmiRs obtained from human miRNA microarray was shown as log_2_ fold-change. **(B)** Levels of miR-155 and miR-146a were determined by qPCR and plotted as relative to that seen in the mock-infected cells. Data were normalized against U6 snRNA. h, hours; JEV, Japanese encephalitis virus; pi, post-infection. *, *P* <0.05; **, *P* <0.005.

In order to understand how JEV infection modulates immune pathogenesis-related miRNAs, we carried out a global human miRNA array study to identify differentially expressed miRNAs in human microglial CHME3 cells in response to JEV infection. Of the several miRNAs modulated during the course of infection, we focused here on a subset of seven miRNAs that have previously been defined as NeurimmiRs [8,9]. Following the bioinformatics and statistical analysis, we found that six of these NeurimmiRs were differentially expressed in CHME3 cells during JEV infection (Figure 1A). We observed that miR-125b and miR-132 remained down-regulated throughout the course of infection. While miR-146a and miR-326 were up-regulated at an early time point (6 hours pi), these were down-regulated as the infection progressed. On the other hand, miR-124 and miR-155 were suppressed at early time points, but were up-regulated at later time points. No change was seen in miR-212 levels during JEV infection.As this study focused on miR146a and miR-155 we validated their expression in human microglial cells by qPCR using the TaqMan assay. The qPCR data generally conformed to the microarray data. Thus, miR-146a expression levels showed a small increase at 6 hours pi followed by a reduction at 24 hours pi. For miR-155 however, the expression levels showed a small decrease at 6 hours pi that was followed by a moderate (approximately 1.8-fold) increase at 48 hours pi (Figure 1B).

### miR-155 suppresses JEV replication in microglial cells

**Figure 2:**
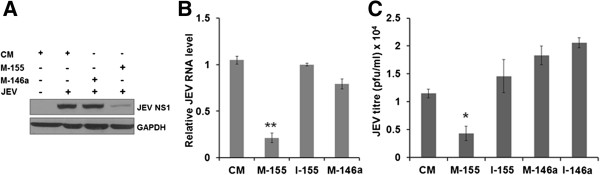
**JEV replication in human microglial cells.** CHME3 cells were transfected with control mimic (CM), mimic-155 (M-155) and mimic-146a (M-146a) followed by JEV infection. Virus replication was monitored at 24 hours pi. **(A)** JEV *NS1* expression was studied using western blotting. **(B)** Relative levels of JEV RNA were quantified by qPCR. Data were normalized against GAPDH RNA. **(C)** JEV titres in culture supernatants were determined by plaque assay. GAPDH, Glyceraldehyde 3-phosphate dehydrogenase; JEV, Japanese encephalitis virus; pi, post-infection. *, *P* <0.05; **, *P* <0.005.

In order to understand the effect of miR-155 and miR-146a modulation on JEV replication, we overexpressed these miRNAs in microglial cells by transfecting specific mimics and infected the cells with JEV. At 24 hours pi, levels of the JEV *NS1* protein and JEV RNA were significantly reduced in miR-155 overexpressing microglial cells, but these were largely unaffected in miR-146a overexpressing cells (Figure 2A and B). A significant suppression of JEV titres (approximately 1 log2) was observed in miR-155 overexpressing cells while miR-146a overexpression had no effect on JEV titre (Figure 2C). These data clearly show that miR-155 had an inhibitory effect on JEV replication in microglial cells.

### miR-155 modulates JEV-induced IFN-β expression in microglial cells

**Figure 3:**
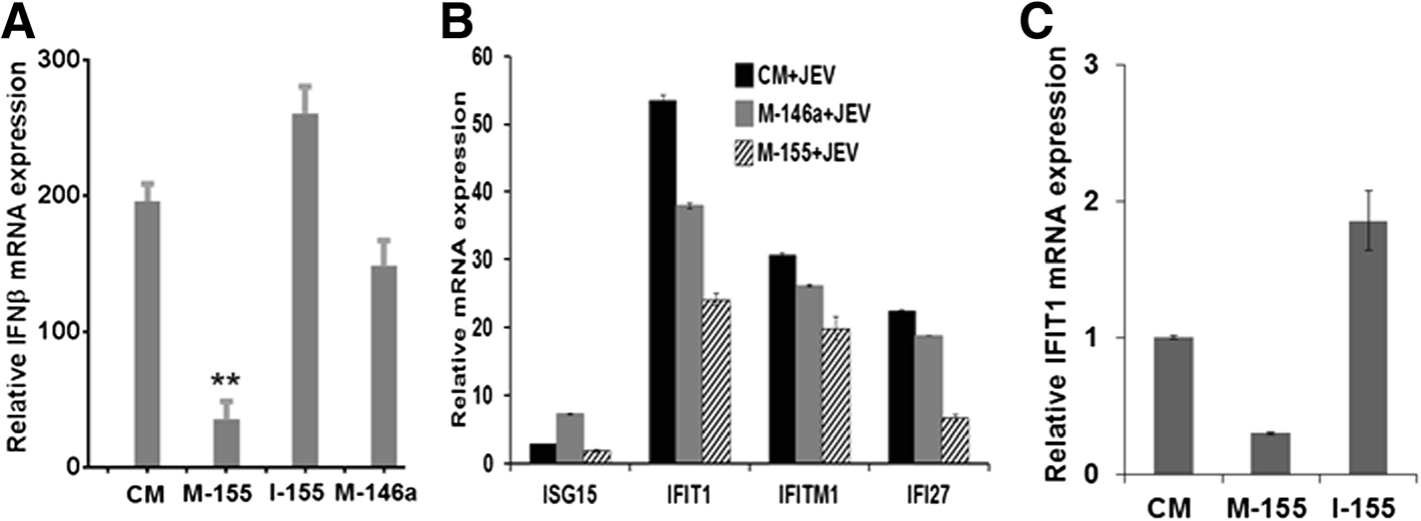
**JEV-induced IFN-β mRNA expression in human microglial cells.** CHME3 cells were transfected with CM, M-155, M-146a, or I-155 and mock-infected or infected with JEV 24 hours later. Total RNA was extracted 24 hours pi. and levels of various transcripts were determined by qPCR normalized against those of *GAPDH*. **(A)** Relative *IFNβ* mRNA expression in JEV-infected cells compared to mock-infected cells. **(B)** Relative abundance of various ISG transcripts in JEV-infected cells compared to mock-infected cells. **(C)** Relative expression of *IFIT1* in M-155 and I-155 transfected cells compared to CM transfected cells following JEV infection. CM, control mimic; GAPDH, Glyceraldehyde 3-phosphate dehydrogenase; I-155, h, hours; inhibitor 155; IFN, interferon; ISG, interferon-stimulated genes; JEV, Japanese encephalitis virus; M-146a, mimic 146a; M-155, mimic 155; pi, post-infection. **, *P* <0.005.

The innate immune response involving the induction of type I IFN constitutes the first line of antiviral defenses and could contribute to reduced virus replication. Since miR-155 and miR-146a are known to modulate IFN-β expression in peripheral organs [15,16], we focused on how these miRNAs affect *IFN-β* mRNA expression in JEV-infected microglial cells. For this, we overexpressed these miRNAs in CHME3 cells by transfecting specific mimic (mimic-155 or mimic-146a) 24 hours prior to JEV infection. Our data showed that JEV-induced *IFN-β* mRNA expression in CHME3 cells and the levels of this JEV-induced *IFN-β* mRNA were significantly reduced in miR-155 overexpressing cells, while only a marginal effect was observed in miR-146a overexpressing cells (Figure 3A). In fact, the suppressive effect of miR-155 on *IFN-β* mRNA expression was reversed when the miR-155 inhibitor was used. Thus cells transfected with the miR-155 inhibitor prior to JEV infection showed an enhanced *IFN-β* mRNA expression when compared to control mimic-transfected cells infected with JEV (Figure 3A).

Infection of mammalian cells by viruses leads to expression of interferon-stimulated genes (ISG) [24]. We have studied the effect of miR-155 and miR-146a overexpression on ISG expression in JEV-infected microglial cells. Figure 3B shows that JEV-induced ISG expression was significantly repressed in miR-155 overexpressing cells, whereas it was only marginally affected in miR-146a overexpressing cells. The suppressive effect of miR-155 overexpression on *IFIT1* expression could be decreased by the miR-155 inhibitor (Figure 3C). These data indicate that JEV infection of miR-155 overexpressing microglial cells resulted in a reduced expression of *IFN-β* and ISG.

### miR-155 attenuates JEV-induced IRF8 expression in microglial cells

**Figure 4:**
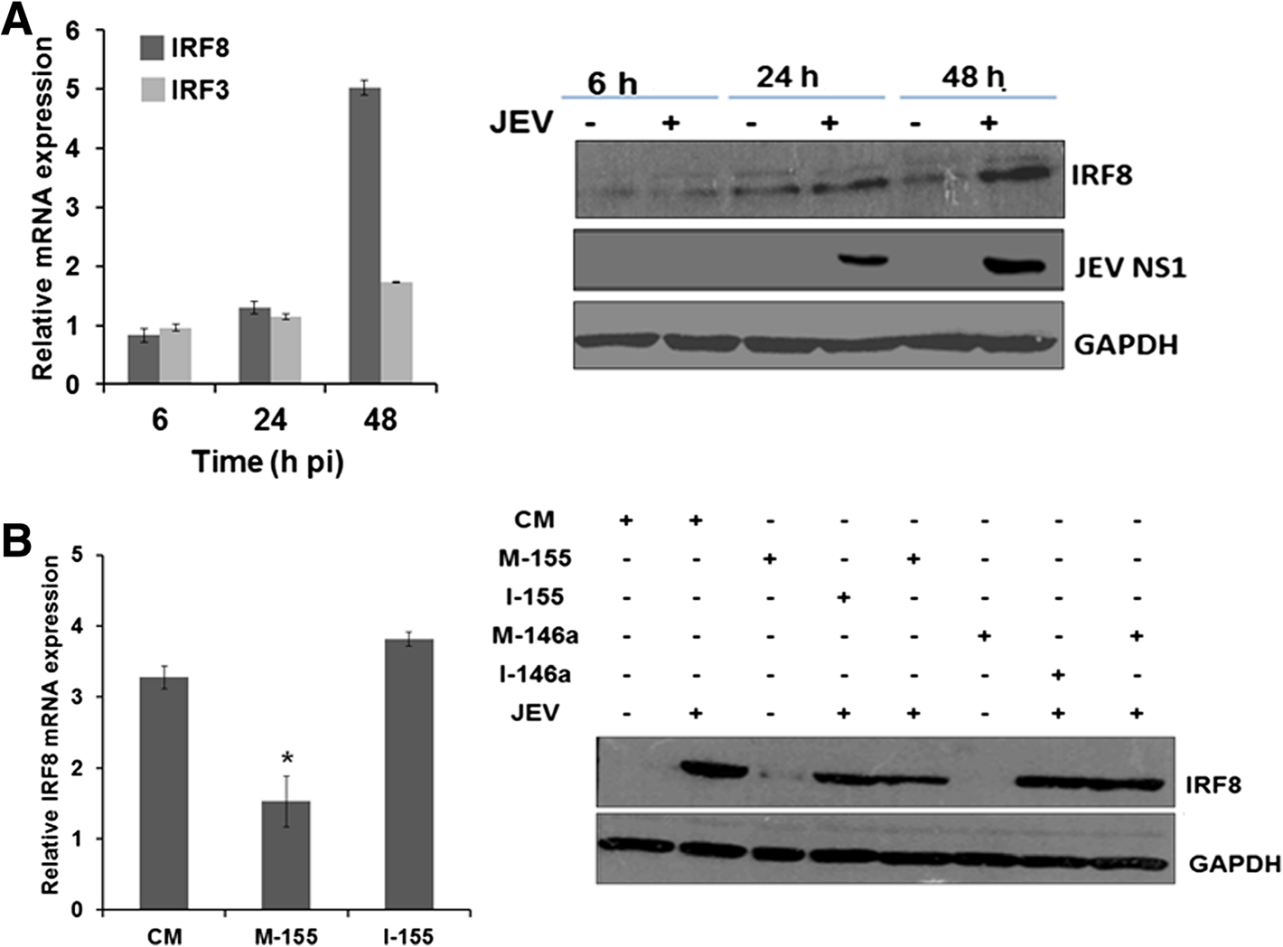
**JEV-induced IRF8 expression in human microglial cells. (A)** CHME3 cells were mock-infected or infected with JEV and total RNA was extracted from cells harvested at different time points. Relative levels of *IRF8* and *IRF3* transcripts in JEV-infected cells compared to those in mock-infected cells are presented in the left panel. GAPDH expression was used to normalize the data. The right panel shows *IRF8* protein levels during JEV infection. **(B)** CHME3 cells were transfected with CM, M-155 and I-155 followed by JEV infection. The left panel shows the relative abundance of *IRF8* measured by qPCR in JEV infected cells in comparison to that in mock-infected cells. GAPDH expression was used to normalize the data. The right panel shows the western blot of *IRF8*. CM, control mimic; GAPDH, Glyceraldehyde 3-phosphate dehydrogenase; h, hours; I-146a, inhibitor 146a; I-155, inhibitor 155; IRF, interferon regulatory factor; JEV, Japanese encephalitis virus; M-146a, mimic 146a; M-155, mimic 155; pi, post-infection. *, *P* <0.05.

The interferon regulatory factor (IRF) family of transcription factors regulate the entire type I IFN system. *IRF8*, a member of IRF family, is a myeloid lineage transcriptional regulator that plays an essential role in the microglial activation and is involved in inducing innate immune response. *IRF8* and *IRF3* can cooperatively induce rapid *IFN-β* production in monocytes [25]. It is also reported that TLR receptor signaling can activate NF-κB through *IRF8* in myeloid lineage cells [26]. We therefore, studied *IRF3* and *IRF8* expression in human microglial cells during JEV infection. Our results suggested that *IRF8* was induced during JEV infection and significantly up-regulated mRNA levels were observed at 48 hours pi. However, there was relatively little increase in *IRF3* expression during JEV infection (Figure 4A, left panel). Western blot results corroborated this finding where enhanced IRF8 protein levels were seen in JEV-infected cells at 48 hours pi (Figure 4A, right panel). Interestingly, JEV-induced *IRF8* expression was suppressed in miR-155 overexpressing cells and this could be reversed using the miR-155 inhibitor (Figure 4B, left panel). Importantly, expression of *IRF8* was not affected in miR146a overexpressing cells (Figure 4B, right panel). These data demonstrate that miR-155 can suppress JEV-induced *IRF8* expression and this might result in the reduced *IFN-ß* levels seen above.

### miR-155 attenuates JEV-induced *NF-κB* pathway gene expression in microglial cells

**Figure 5:**
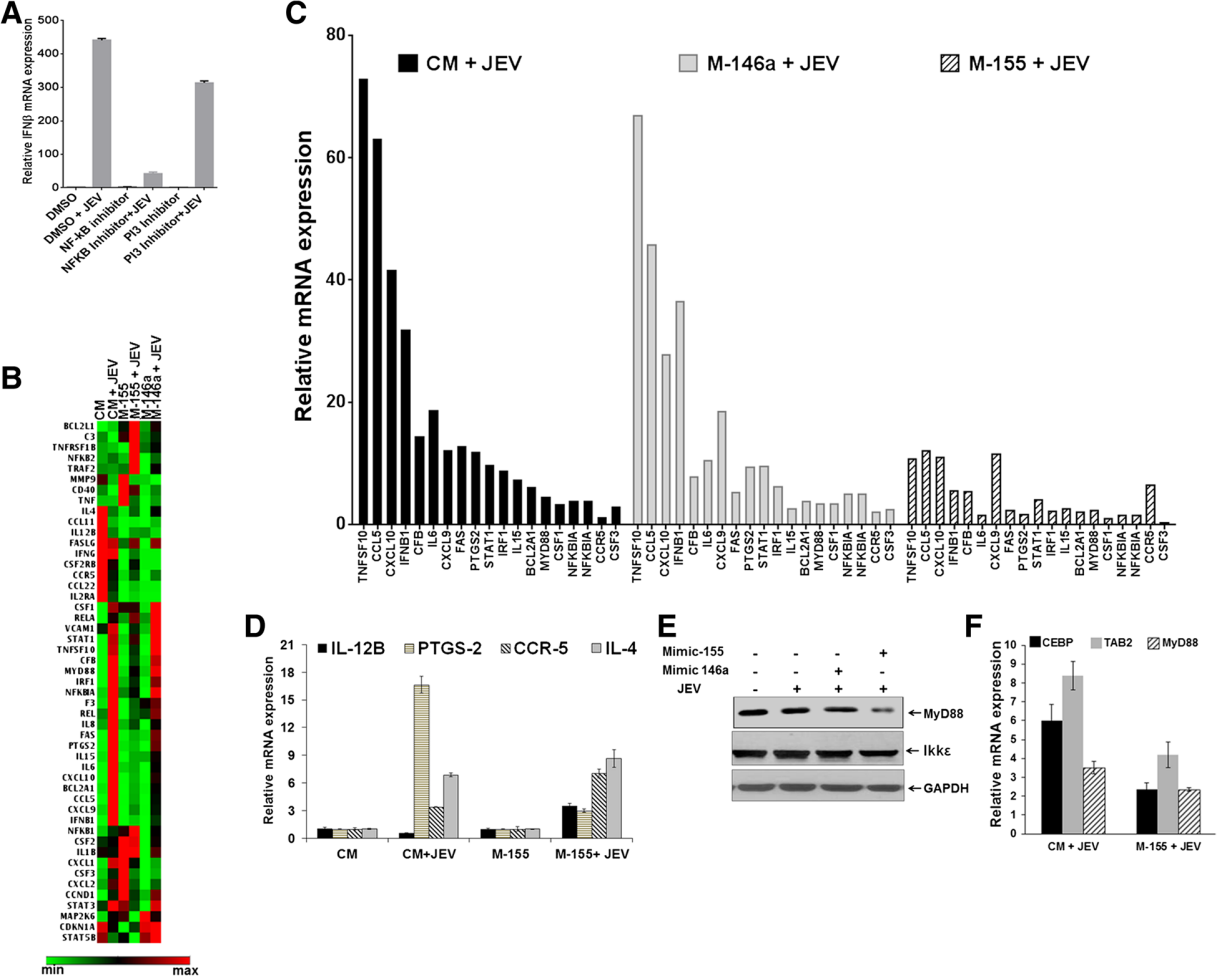
**JEV-induced NF-κB targeted gene expression in human microglial cells. (A)** CHME3 cells were treated for 6 hours with NF-κB inhibitor (25 μM) or PI3K inhibitor (10 μM) using DMSO as a vehicle. This was followed by JEV infection for 24 hours. Total RNA was extracted and relative *IFN-β* mRNA expression was measured using qPCR in inhibitor-treated cells compared to the vehicle-treated cells. GAPDH was used to normalize the data. **(B)** CHME3 cells were transfected with CM, M-155 or M-146a and infected with JEV 24 hours later. Total RNA was extracted 24 hours pi and levels of various NF-κB targeted genes were studied using PCR array. Hierarchical clustering represents the co-regulated genes across the groups. Relative gene expression levels were depicted according to the colour scale (green - downregulation, red - upregulation). Gene designations are listed to the left. **(C)** Expression of *NF-κB* targeted genes expression that were found to be common (n = 19) in PCR array among three different groups (CM + JEV, M-146a + JEV, and M-155 + JEV) in CHME3 cells. An attenuated expression of JEV-induced genes was observed in miR-155 overexpressing cells. **(D)** Expression of *IL-12B, PTGS-2, CCR5* and *IL-4* was studied by qPCR. The figure shows a relative abundance of transcripts in JEV-infected, mimic-transfected CHME3 cells when compared with those in cells transfected with the mimics. GAPDH was used to normalize the data. **(E)** Expression of *MyD88* and *Ikkϵ* in miR-155 or miR-146a overexpressing JEV-infected CHME3 cells as seen by western blotting. **(F)** Expression of different mRNAs in miR-155 overexpressing CHME3 cells following infection with JEV. CM, control mimic; DMSO, Dimethyl sulfoxide; GAPDH, Glyceraldehyde 3-phosphate dehydrogenase; IFN, interferon; JEV, Japanese encephalitis virus; M-146a, mimic 146a; M-155, mimic 155.

*NF-κB*-mediated pro-inflammatory gene expression plays an important role in innate immune response against viral infection. Figure 5A shows that *NF-κB* activation is required for *IFN-β* induction in JEV-infected cells as inhibition of *NF-κB* activity led to reduced IFN-β mRNA expression in human microglial cells. Since JEV-induced *IFN-β* expression was suppressed in miR-155 overexpressing cells (Figure 3A), we hypothesized that miR-155 might attenuate JEV-induced innate immune gene expression. In order to identify the innate immune genes that are modulated by miR-155 during JEV infection, a PCR array was performed covering 84 genes directly or indirectly regulated via the *NF-κB* pathway. The heat map shown in Figure 5B, depicts dysregulated genes in CHME3 cells during JEV infection. Cells transfected with the control mimic followed by JEV infection had 26 genes up-regulated (over 2-fold). On the other hand, 32 genes were up-regulated in miR-155 overexpressing cells infected with JEV. Interestingly, 19 of these up-regulated genes were common in both sets. However, 16 out of these 19 genes exhibited attenuation of their expression in miR-155 overexpressing JEV-infected cells (Figure 5C). We studied the expression of several of these mRNAs by qPCR (Figure 5D). *IL-12B* and *IL-4* are anti-inflammatory genes and the expression of these was up-regulated whereas *PTGS2 (COX2),* an inflammatory mediator, was significantly suppressed in miR-155 overexpressing cells. *CCR-5*, which has an important role in JEV infection, [27] was up-regulated in these cells. These qPCR data conformed to the PCR array data.

Interestingly, miR-155 and miR-146a are reported to be involved in the reduction of *NF-κB* activity by targeting specific genes (*Ikkϵ, MyD88, TAB2, TRAF6, IRAK1*) in the TLR-mediated *NF-κB* activating pathways [15,28]. PCR array data showed that JEV-induced MyD88 mRNA expression was suppressed in miR-155 overexpressing cells. The reduced MyD88 protein level was confirmed by western blot. However, *Ikkϵ* protein levels were not affected (Figure 5E). As observed in the PCR array, *IL-6* mRNA expression was markedly reduced in M-155 overexpressing cells following JEV infection. Transcription factor C/EBPβ, which activates IL-6 gene expression, is among the potential direct mRNA targets of miR-155. TGF-ß activated kinase 1/MAP3K7 binding protein 2 (*TAB2*) is another important multifunctional molecule that activates NF-κB. Our results showed that both *C/EBPβ* and *TAB2* mRNA expression were significantly attenuated in miR-155 overexpressing cells following JEV infection (Figure 5F). These results indicate that miR-155 can differentially target multiple genes in the *NF-κB* signaling pathway, resulting in significant modulation of the expression of innate immune genes in JEV-infected human microglial cells.

### miR-155 enhances JEV-induced *CD45* expression on microglial cells

**Figure 6:**
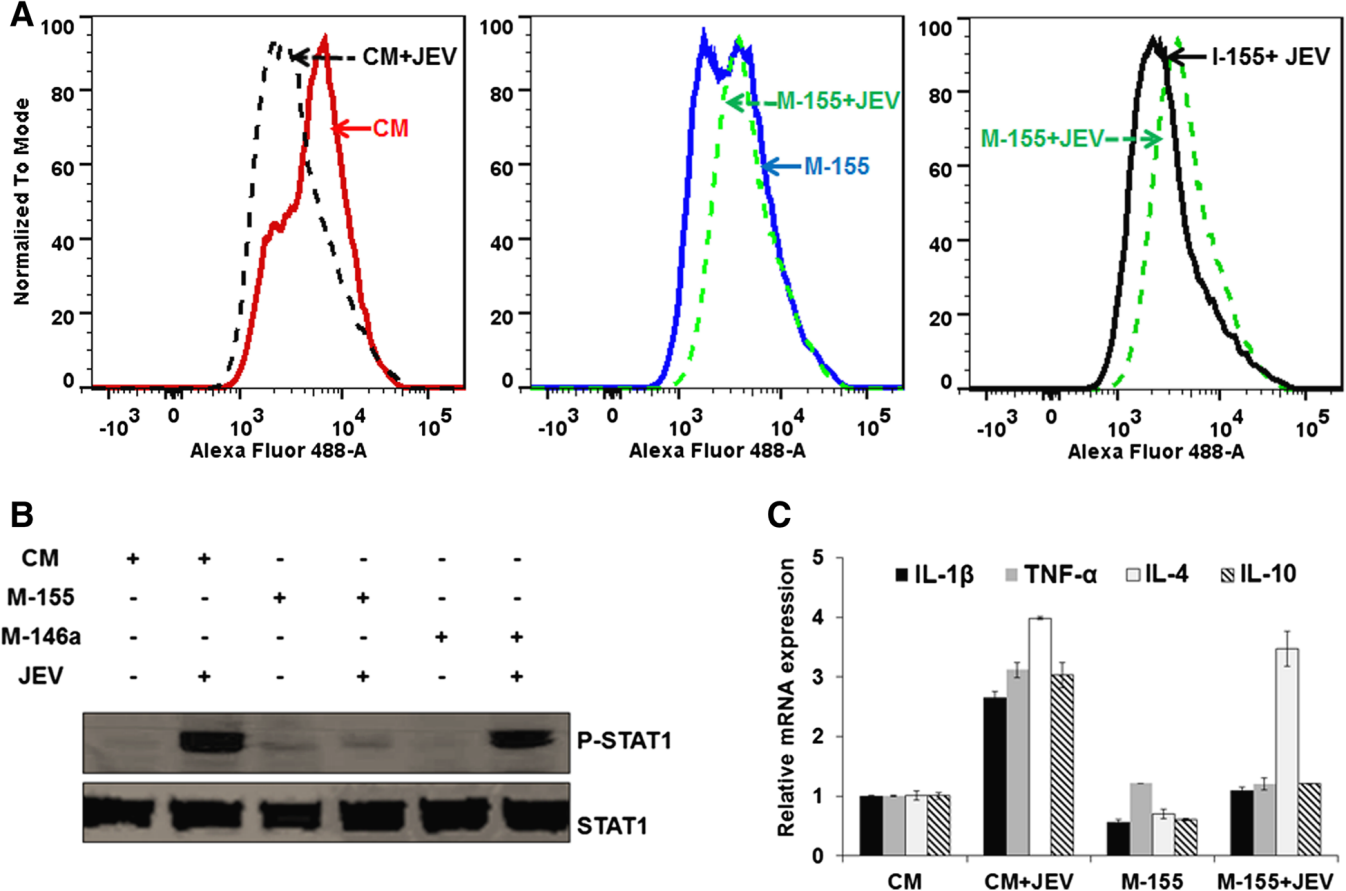
**JEV-induced *CD45 *expressions in human microglial cells. (A)** CHME3 cells were transfected with CM, M-155 or I-155 followed by JEV infection for 24 hours. Cells were harvested and examined for *CD45* expression by FACS. Data were analyzed by FlowJo software (Tree Star). Y-axis represents the number of events normalized according to FlowJo algorithms. **(B)***p-STAT1* and *STAT1* expression was studied in JEV-infected cells by western blotting. **(C)** Relative expression of *TNF-α, IL-1β, IL-4* and *IL10* mRNA in M-155 transfected cells infected with JEV when compared with those transfected with CM. The data were normalized against GAPDH. CM, control mimic; FACS, fluorescence activated cell sorter; GAPDH, Glyceraldehyde 3-phosphate dehydrogenase; I-155, inhibitor 155; JEV, Japanese encephalitis virus; M-146a, mimic 146a; M-155, mimic 155.

Following an infection or an injury, resting microglial cells could polarize into two major activated subtypes categorized as M1 and M2. The M1 subtype overproduces pro-inflammatory cytokines and promotes cell-mediated immunity, whereas M2 microglia tends to dampen inflammation and clear cellular debris [29]. In this context *CD45*, a hematopoietic cell-specific protein tyrosine phosphatase, acts as a negative regulator of cytokine receptor signaling leading to altered microglial activation [29-31]. We observed that JEV infection in miR-155 overexpressing human microglial cells up-regulated *CD45* expression on cell surface (Figure 6A). As a downstream effect of this modulation, we have checked *p-STAT1* expression (which is important in pro-inflammatory cytokine production) as well as the expression of ISG. We observed that JEV infection in the control mimic or miR-146a overexpressing cells led to enhanced *p-STAT1* expression. On the other hand, reduced p-*STAT1* expression was observed in miR-155 overexpressing cells infected with JEV (Figure 6B). We have also examined the mRNA expression of other cytokine genes by qPCR. Thus, *IL-1β, TNF-α,* and *IL-10* expression was significantly reduced in miR-155 overexpressing JEV-infected cells, but *IL-4* levels were not affected (Figure 6C). Together, these data show that overexpression of miR-155 during JEV infection enhances *CD45* expression on the microglial cell surface and negatively modulates cytokine expression.

### miR-155 suppresses JEV-induced complement factor H expression in microglial cells

**Figure 7:**
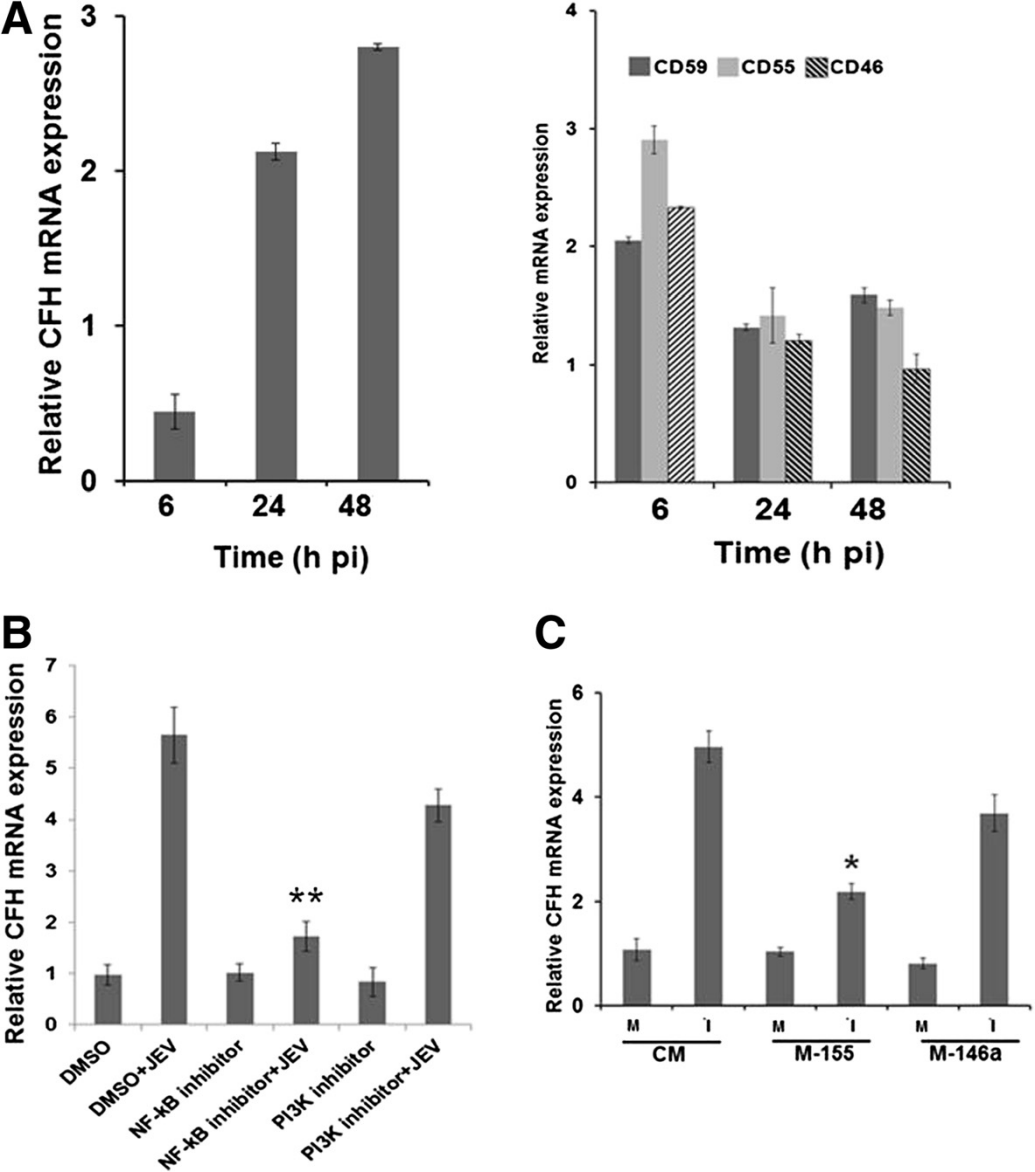
**JEV-induced CFH expression in human microglial cells. (A)** CHME3 cells were mock-infected or infected with JEV. Total RNA was extracted from cells at different time points. The relative mRNA levels of *CFH* (left panel) and *CD59*, *CD55*, and *CD46* (right panel) were determined in JEV-infected cells using qPCR in comparison to that in mock-infected cells. *GAPDH* was used as the normalizing control. **(B)** CHME3 cells were treated with either DMSO as a vehicle, *NF-κB* (25 μM) or PI3K inhibitor (10 μM) in DMSO for 6 hours followed by JEV infection for 24 hours. Total RNA was extracted and relative levels of *CFH* expression was studied by qPCR in different treatments when compared with vehicle-treated cells. **(C)** CHME3 was transfected with CM, M-155 or M-146a. After 24 hours of transfection cells were either mock-infected or infected with JEV for another 24 hours. Total RNA was extracted and relative levels of *CFH* mRNA determined in JEV-infected cells in comparison to those seen in mock-infected cells. CFH, complement factor H; CM, control mimic; DMSO, Dimethyl sulfoxide; *GAPDH*, Glyceraldehyde 3-phosphate dehydrogenase; h, hours; JEV, Japanese encephalitis virus; M-146a, mimic 146a; M-155, mimic 155; pi, post-infection. *, *P* <0.05; **, *P* <0.005.

Complement factor H (*CFH*) is a central regulator of the complement system and viruses could up-regulate *CFH* expression to inhibit excessive complement activation against them. *CFH* expression can be regulated by miR-155 or miR-146 as both of them have matched seed sequences on the 3’-untranslated region (UTR) of the *CFH* gene [17,18]. In this study, we examined the expression of CFH and other complement pathway regulators in microglial cells following JEV infection. We found that CFH mRNA expression was significantly enhanced at 48 hours pi (Figure 7A, left panel). Expression of other complement regulators (*CD46*, *CD55*, or *CD59*) showed a marginal increase during the early phase of JEV infection (6 hours pi) but at the later time points it was same as in uninfected control cells (Figure 7A, right panel). Moreover, JEV-induced CFH expression was *NF-κB* dependent as *NF-κB* inhibition prior to JEV infection did not enhance *CFH* expression (Figure 7B). Further, overexpression of miR-155, but not miR-146a, attenuated the CFH expression in microglial cells during JEV infection (Figure 7C).

## Discussion

Microglia plays key roles in both innate and adaptive immune response in the CNS. In the mammalian immune system miRNAs control differentiation as well as innate and adaptive immune responses. A subset of these miRNAs (designated NeurimmiRs) notably affects both immune and neuronal functions. In this study, we have focused on two NeurimmiRs, miR-155 and miR-146a, and have studied their role in JEV replication and microglial activation during the infection. These two miRNAs are widely reported to have an immense effect on innate immune response in the context of various external stimuli (such as TLR ligands, virus, bacteria, and other microorganisms). However, very little is known about their role in innate immune modulation during neurotropic flavivirus infection. Here, we have used cultured human microglial cells to understand the effect of JEV infection on these two miRNAs. We have also studied how overexpression of these two miRNAs in microglial cells could affect JEV replication and how these miRNAs could modulate innate immune responses as well as microglial activation. We observed that the expression of miR-155 was enhanced in microglial cells during JEV infection. This may be due to the fact that miR-155 production is transiently under the control of *NF-κB* which is activated during JEV infection and inflammation [28,32]. Since both these miRNAs are known to have a role in antiviral immunity, we hypothesized that upregulation of these miRNAs might play a role in controlling JEV infection. Interestingly, we found that JEV replication was significantly restricted in miR-155 overexpressing cells.

To gain an insight into the mechanism of how miR-155 overexpression suppressed JEV replication, we analyzed JEV genomic RNA *in silico* with miRNA target prediction algorithms (PITA and RNA22) but found no potential target sites for miR-155. Hence, miR-155 is unlikely to target JEV RNA directly. We next examined *IFN*-β in JEV-infected miRNAs overexpressing cells as inducible miR-155 in response to the vesicular stomatitis virus (an RNA virus) infection in macrophage was shown to induce type I IFN signaling and inhibit viral replication [16]. However, to our surprise we found significant reduction in JEV-induced *IFN-β* as well as downstream ISG mRNA expression in cells where miR-155 was overexpressed. This suggests that miR-155 uses different mechanisms to exert an antiviral effect against JEV in microglial cells. In fact, Swaminathan *et al.*[21] have shown that miR-155 exerts an anti-HIV-1 effect by targeting several HIV-1 dependency factors involved in post-entry, pre-integration events, leading to severely diminished HIV-1 infection. Thus, miR-155 can use different mechanisms for its antiviral effect depending upon the virus and the environmental stimuli generated during the particular virus infection of a certain cell type.

In order to understand reduced *IFN*-β expression in JEV-infected miR-155 overexpressing cells, we checked the expression of IRFs. Among the several IRFs reported, we focused our study on *IRF8* as it plays a major role in *IFN* signaling, response to infection, and maturation of myeloid lineages cells [33]. *IRF8* is also involved in the rapid induction of *IFN*-β in human monocytes [25]. Moreover, *IRF8* may activate a program of gene expression that transforms microglia into a reactive phenotype [34]. In this study, we observed that JEV infection in microglia can induce *IRF8* expression, but the same expression was attenuated in miR-155 overexpressing cells and may be a possible reason for reduced *IFN*-β production in miR-155 overexpressing cells. Interestingly, bioinformatics analysis predicted IRF8 as one of the potential targets for miR-155. We cloned 3’-UTR of *IRF8* in the luciferase reporter system for miRNA target validation but found no change in the luciferase readout in miR-155 overexpressing cells (data not shown). We have shown that *IRF8* is induced during JEV infection. Since JEV replication and titres are reduced in miR-155 overexpressing cells, it could result in reduced *IRF8* induction.

*IRF8* is also involved in TLR-mediated *NF-κB* activation [26]. Since JEV-induced *IRF8* expression in miR-155 overexpressing cells was attenuated, we sought to understand its effect on *NF-κB* pathway. For this we examined 84 genes by RT-PCR array whose expression and function, either directly or indirectly, depend on *NF-κB* activation. We found several genes, including *IFN*-β, *MyD88, STAT1, PTGS2*, and *IL-12B*, which are induced by JEV and show attenuated expression in miR-155 overexpressing cells. Recently, *CCR5* receptor expression in a mouse model of JE was reported to play an important role in recovery as well as promote host survival against JEV [27]. Interestingly, increased *CCR5* expression was also observed in our cell culture based study. Thus, modulation of NF-κB mediated signaling pathway genes by JEV-induced miR-155 expression might play a role in reduced JEV replication in microglial cells.

Specific induction of active SHP2 phosphatase dephosphorylates IRF8, which in turn becomes an active repressor and down-regulates TLR-mediated gene expression [35]. In hematopoietic cell lineage *CD45* (a Src-homology 2 domain (SH2)-containing protein tyrosine phosphatase) plays an important role in regulating cytokine receptor mediated signal [36]. Analysis of microglia *ex vivo* revealed that IRF8-deficient microglia had significantly increased levels of *CD45*[37]. Thus attenuated *IRF8* expression in miR-155 overexpressing cells may result in enhanced *CD45* expression on microglial cells.

Following an infection, resting microglial cells can get activated into M1 or M2 phenotypes. The M1 phenotype is a pro-inflammatory state and induces neuropathology, whereas M2 is anti-inflammatory state that could have a neuroprotective role [36]. It is reported that *CD45* can negatively regulate CD40L-CD40-induced microglial M1 activation; an effect leading to the promotion of the M2 phenotype. Moreover, this *CD45*-mediated activation state appears to decrease harmful cytokine production [36]. In this study, we observed an increased *CD45* expression in JEV-infected miR-155 overexpressing cells and relatively reduced *p-STAT1* expression. Further, mRNA expression for pro-inflammatory cytokines *IL-1β* and *TNF-α* was reduced in miR-155 overexpressing cells. *CD45* has also been shown to down-regulate NF-κB, an important mediator of pro-inflammatory cytokines [38]. Thus, increased *CD45* expression in JEV-induced miR-155 expressing cells may explain reduced expression of NF-κB pathway genes in our study. Taken together, these data show that miR-155 induction can modulate the JEV-induced microglial activation to a state that may be beneficial to the host. Further *in vivo* study is needed to clarify this issue.

Complement activation is considered to be an important component of innate immune response against invading pathogens. *CFH* is one of the regulators which negatively regulate the complement activation. Viruses can utilize *CFH* to evade the innate immune response. The non-structural protein NS1 of the West Nile virus, a flavivirus, inhibits complement activation by binding to *CFH*[39]. *CFH* can be induced by *NF-κB* activation and can be regulated through miRNAs [17]. Interestingly, both miR-155 and miR-146a have the same target sequence in the 3’-UTR of *CFH* and this has been experimentally proven for both the miRNAs [17]. We have shown that JEV infection can induce *CFH* expression in human and mouse microglial cells and this induction is attenuated in miR-155 overexpressing cells. Therefore, it is possible that miR-155 expression in JEV-infected microglial cells inhibits *CFH* expression, which in turn may benefit the host by facilitating complement activation against JEV.

In a study published earlier this month, Thounaojam *et al*. [40] showed an up-regulation of miR-155 in mouse microglial cells (BV-2) and in the mouse and human brain during JEV infection. They suggested that miR-155 had a pro-inflammatory role as its inhibition decreased *TBK-1, IRF3/7*, and *NF-κB* phosphorylation both in BV-2 cells as well as in the mouse brain. These results point to a role for miR-155 that is opposite to what we have observed in our study. A major difference between these two studies is that while results reported by Thounaojam *et al.*[40] are derived from mouse BV-2 cells and the mouse brain where various different kinds of cells may get infected with JEV, our results are derived entirely from *in vitro* cultured human microglial cells CHME3. Additionally, the GP78 strain of JEV used by Thounaojam *et al.*[40] is a slow growing virus, both in cultured cells and the mouse brain, with lesions in the virus-cell fusion process [22]. Another reason for these differences may be related to the smaller increase (approximately 4-fold) of miR-155 during JEV infection in BV2 cells [40], compared with the super maximal concentration in CHME3 cells (approximately 100-fold) that occurred during miR-155 overexpression in the present study. Several reports have suggested that, depending on the expression level, miR-155 can modulate cellular functions by targeting genes in different pathways. Ceppi *et al.*[41] showed that low level miR-155 expression enables the activation of *p38 MAPK* pathway, favoring *IL-1β* expression, which induces inflammation in an autocrine manner. However, the same pathway was inhibited when the miR-155 expression level went up significantly, ultimately reflecting an altered immune profile. Similar observations were made by Xiao *et al.*[42] who reported a 3-fold induction of miR-155 and pro-inflammatory cytokine responses during *Helicobacter pylori* infection, whereas the overexpression of miR-155 negatively regulated the pro-inflammatory responses.

Intracellular signaling pathways that are concomitantly activated by the same stimulus often interact with one another through a cross regulatory feedback mechanism. The miR-155 is a multifunctional microRNA and it can modulate inflammatory responses in both a positive and negative way [40-48]. Besides its positive role in *NF-κB* activation and subsequent pro-inflammatory response, accumulating evidence has demonstrated that it can constitute a negative feedback loop in the *NF-κB* signaling pathway by targeting multiple key proteins, which ultimately leads to repression of, or at least the limitation of *NF-κB* activation in response to viral or microbial stimuli [41,42,45-48]. Therefore, miR-155 could modulate inflammation depending on various factors including its expression level, the cell type, and the environmental stimuli.

Overall our study suggests that miR-155 modulation can act on multiple levels to control JEV infection of microglial cells and induce innate immune responses that may be beneficial to the host. It can enhance *CD45* expression, reduce pro-inflammatory cytokines and *CFH* expression by targeting several key genes, and suppress JEV replication in microglial cells. These data point to miR-155 playing an important role in modulating JEV-induced microglial activation that may be beneficial in limiting JEV infection in the host. Additional studies, both *in vitro* and *in vivo*, are needed to further understand the role of miR-155 during JEV infection in switching microglial activation towards the neuroprotective state.

## Abbreviations

ANOVA: Analysis Of Variance; CCR5: C-C Chemokine Receptor type 5; CEBPβ: CCAAT/Enhancer Binding Protein beta; CFH: Complement Factor H; CNS: Central Nervous System; COX-2: Cyclooxygenase-2; DENV 2: Dengue Virus 2; DMEM: Dulbecco’s Modified Eagle’s Medium; DMSO: Di Methyl Sulfoxide; FACS: Fluorescence Activated Cell Sorter; FBS: Fetal Bovine Serum; GAPDH: Glyceraldehyde 3-Phosphate Dehydrogenase; HIV: Human Immunodeficiency Virus; HRP: Horseradish Peroxidase; IRF: Interferon Regulatory Factor; ISG: Interferon Stimulated Genes; JE: Japanese Encephalitis; JEV: Japanese Encephalitis Virus; LPS: Lipopolysaccharide; MEM: Minimal Essential Medium; MOI: Multiplicity Of Infection; MyD88: Myeloid Differentiation primary response gene (88); NF-kB: Nuclear Factor kappa light chain enhancer of activated B cells; NFQ-MGB: Non fluorescent Quencher-Minor Groove Binder; PCR: Polymerase Chain Reaction; PFU: Plaque Forming Unit; PI3K: Phosphatidylinositol-4,5-bisphosphate 3-kinase; Poly (I:C): Polyinosinic-polycytidylic acid; PS: Porcine Stable kidney cell line; p-STAT1: Phosphorylated-Signal Transducers and Activators of Transcription; PTGS-2: Prostaglandin-endoperoxide Synthase 2; qPCR: Quantitative real-time PCR; SDS-PAGE: Sodium Dodecyl Sulfate-Polyacrylamide Gel; STAT1: Signal Transducers and Activators of Transcription; TBK1: TANK Binding Kinase 1; TLR: Toll Like Receptor; TNF-α: Tumor Necrosis Factor-alpha.

## Competing interests

The authors declare that they have no competing interests.

## Authors’ contributions

AB was responsible for experimental design, data analysis, and drafting the manuscript. SP, BK, and PJ performed the RNA extraction, PCR array, RT-PCR, miRNA assay, western blot, and FACS. SR and BK performed the virus preparation, cell culture, and transfection and virus infection experiments. SV and AB conceived the idea, supervised the experiments, and participated in editing the manuscript. All authors have read and approved the final manuscript.

## References

[B1] Thongtan T Cheepsunthorn P Chaiworakul V Rattanarungsan C Wikan N Smith DR Highly permissive infection of microglial cells by Japanese encephalitis virus: a possible role as a viral reservoir Microbes Infect 2010 12 37 45 10.1016/j.micinf.2009.09.013 19786116

[B2] Chen CJ Ou YC Lin SY Raung SL Liao SL Lai CY Chen SY Chen JH Glial activation involvement in neuronal death by Japanese encephalitis virus infection J Gen Virol 2010 91 1028 1037 10.1099/vir.0.013565-0 20007359

[B3] Das S Dutta K Kumawat KL Ghoshal A Adhya D Basu A Abrogated inflammatory response promotes neurogenesis in a murine model of Japanese encephalitis PLoS One 2011 6 e17225 10.1371/journal.pone.0017225 21390230 PMC3048396

[B4] Kobayashi K Imagama S Ohgomori T Hirano K Uchimura K Sakamoto K Hirakawa A Takeuchi H Suzumura A Ishiguro N Kadomatsu K Minocycline selectively inhibits M1 polarization of microglia Cell Death Dis 2013 4 e525 10.1038/cddis.2013.54 23470532 PMC3613832

[B5] Mishra MK Basu A Minocycline neuroprotects, reduces microglial activation, inhibits caspase 3 induction, and viral replication following Japanese encephalitis J Neurochem 2008 105 1582 1595 10.1111/j.1471-4159.2008.05238.x 18208541

[B6] Ponomarev ED Veremeyko T Weiner HL MicroRNAs are universal regulators of differentiation, activation, and polarization of microglia and macrophages in normal and diseased CNS Glia 2013 61 91 103 10.1002/glia.22363 22653784 PMC3434289

[B7] Skalsky RL Cullen BR Viruses, microRNAs, and host interactions Annu Rev Microbiol 2010 64 123 141 10.1146/annurev.micro.112408.134243 20477536 PMC3621958

[B8] Soreq H Wolf Y NeurimmiRs: microRNAs in the neuroimmune interface Trends Mol Med 2011 17 548 555 10.1016/j.molmed.2011.06.009 21813326

[B9] Wanet A Tacheny A Arnould T Renard P miR-212/132 expression and functions: within and beyond the neuronal compartment Nucleic Acids Res 2012 40 4742 4753 10.1093/nar/gks151 22362752 PMC3367188

[B10] Lindsay MA microRNAs and the immune response Trends Immunol 2011 29 343 351 18515182 10.1016/j.it.2008.04.004

[B11] Elton TS Selemon H Elton SM Parinandi NL Regulation of the MIR155 host gene in physiological and pathological processes Gene 2013 532 1 12 10.1016/j.gene.2012.12.009 23246696

[B12] O’Connell RM Rao DS Chaudhuri AA Baltimore D Physiological and pathological roles for microRNAs in the immune system Nat Rev Immunol 2010 10 111 122 10.1038/nri2708 20098459

[B13] Cardoso AL Guedes JR Pereira de Almeida L Pedroso de Lima MC miR-155 modulates microglia-mediated immune response by down-regulating SOCS-1 and promoting cytokine and nitric oxide production Immunology 2012 135 73 88 10.1111/j.1365-2567.2011.03514.x 22043967 PMC3246654

[B14] Saba R Gushue S Huzarewich RLCH Manguiat K Medina S Robertson C Booth SA MicroRNA 146a (miR-146a) is overexpressed during prion disease and modulates the innate immune response and the microglial activation state PLoS One 2012 7 e30832 10.1371/journal.pone.0030832 22363497 PMC3281888

[B15] Hou J Wang P Lin L Liu X Ma F An H Wang Z Cao X MicroRNA-146a feedback inhibits RIG-I-dependent Type I IFN production in macrophages by targeting TRAF6, IRAK1, and IRAK2 J Immunol 2009 183 2150 2158 10.4049/jimmunol.0900707 19596990

[B16] Wang P Hou J Lin L Wang C Liu X Li D Ma F Wang Z Cao X Inducible microRNA-155 feedback promotes type I IFN signaling in antiviral innate immunity by targeting suppressor of cytokine signaling 1 J Immunol 2010 185 6226 6233 10.4049/jimmunol.1000491 20937844

[B17] Lukiw WJ Surjyadipta B Dua P Alexandrov PN Common micro RNAs (miRNAs) target complement factor H (CFH) regulation in Alzheimer’s disease (AD) and in age-related macular degeneration (AMD) Int J Biochem Mol Biol 2012 3 105 116 22509485 PMC3325769

[B18] Lukiw WJ Alexandrov PN Regulation of complement factor H (CFH) by multiple miRNAs in Alzheimer’s disease (AD) brain Mol Neurobiol 2012 46 11 19 10.1007/s12035-012-8234-4 22302353 PMC3703615

[B19] Li YY Alexandrov PN Pogue AI Zhao Y Bhattacharjee S Lukiw WJ miRNA-155 upregulation and complement factor H deficits in Down’s Syndrome Nuroreport 2012 23 168 173 10.1097/WNR.0b013e32834f4eb4 PMC3264826 22182977

[B20] Wu S He L Li Y Wang T Feng L Jiang L Zhang P Huang X miR-146a facilitates replication of dengue virus by dampening interferon induction by targeting TRAF6 J Infect 2013 67 329 341 10.1016/j.jinf.2013.05.003 23685241

[B21] Swaminathan G Rossi F Sierra LJ Gupta A Navas-Martín S Martín-García J A role for microRNA-155 modulation in the anti-HIV-1 effects of Toll-like receptor 3 stimulation in macrophages PLoS Pathog 2012 8 e1002937 10.1371/journal.ppat.1002937 23028330 PMC3447756

[B22] Vrati S Agarwal V Malik P Wani SA Saini M Molecular characterization of an Indian isolate of Japanese encephalitis virus that shows an extended lag phase during growth J Gen Virol 1999 80 1665 1671 10423134 10.1099/0022-1317-80-7-1665

[B23] Kalia M Khasa R Sharma M Nain M Vrati S Japanese encephalitis virus infects neuronal cells through a clathrin-independent endocytic mechanism J Virol 2013 87 148 157 10.1128/JVI.01399-12 23055570 PMC3536362

[B24] El-Ekiaby N Hamdi N Negm M Ahmed R Zekri AR Esmat G Abdelaziz AI Repressed induction of interferon-related microRNAs miR-146a and miR-155 in peripheral blood mononuclear cells infected with HCV genotype 4 FEBS Open Bio 2012 2 179 186 23650597 10.1016/j.fob.2012.07.005 PMC3642156

[B25] Li P Wong JJ Sum C Sin WX Ng KQ Koh MB Chin KC IRF8 and IRF3 cooperatively regulate rapid interferon-β induction in human blood monocytes Blood 2011 117 2847 2854 10.1182/blood-2010-07-294272 21228327

[B26] Tsujimura H Tamura T Kong HJ Nishiyama A Ishii KJ Klinman DM Ozato K Toll-like receptor 9 signaling activates NF-kappaB through IFN regulatory factor-8/IFN consensus sequence binding protein in dendritic cells J Immunol 2004 172 6820 6827 10.4049/jimmunol.172.11.6820 15153500

[B27] Larena M Regner M Lobigs M The chemokine receptor CCR5, a therapeutic target for HIV/AIDS antagonists. Is critical for recovery in a mouse model of Japanese encephalitis PLoS One 2012 7 e44834 10.1371/journal.pone.0044834 23028638 PMC3448613

[B28] Boldin MP Baltimore D MicroRNAs, new effectors and regulators of NF-κB Immunol Rev 2012 246 205 220 10.1111/j.1600-065X.2011.01089.x 22435557

[B29] Town T Nikolic V Tan J The microglial “activation” continuum: from innate to adaptive responses J Neuroinflammation 2005 2 24 10.1186/1742-2094-2-24 16259628 PMC1298325

[B30] Irie-Sasaki J Sasaki T Matsumoto W Opavsky A Cheng M Welstead G Griffiths E Krawczyk C Richardson CD Aitken K Iscove N Koretzky G Johnson P Liu P Rothstein DM Penninger JM CD45 is a JAK phosphatase and negatively regulates cytokine receptor signaling Nature 2001 409 349 354 10.1038/35053086 11201744

[B31] Hermiston ML Xu Z Weiss A CD45: a critical regulator of signaling thresholds in immune cells Annu Rev Immunol 2003 21 107 137 10.1146/annurev.immunol.21.120601.140946 12414720

[B32] Cremer TJ Fatehchand K Shah P Gillette D Patel H Marsh RL Besecker BY Rajaram MV Cormet-Boyaka E Kanneganti TD Schlesinger LS Butchar JP Tridandapani S MiR-155 induction by microbes/microbial ligands requires NF-κB-dependent de novo protein synthesis Front Cell Infect Microbiol 2012 2 73 22919664 10.3389/fcimb.2012.00073 PMC3417573

[B33] Berghout J Langlais D Radovanovic I Tam M MacMicking JD Stevenson MM Gros P *Irf8*-regulated genomic responses drive pathological inflammation during cerebral malaria PLoS Pathog 2013 9 e1003491 10.1371/journal.ppat.1003491 23853600 PMC3708918

[B34] Horiuchi M Wakayama K Itoh A Kawai K Pleasure D Ozato K Itoh T Interferon regulatory factor 8/interferon consensus sequence binding protein is a critical transcription factor for the physiological phenotype of microglia J Neuroinflammation 2012 9 227 10.1186/1742-2094-9-227 23020843 PMC3546867

[B35] Fragale A Stellacci E Ilari R Remoli AL Lanciotti A Perrotti E Shytaj I Orsatti R Lawrence HR Lawrence NJ Wu J Rehli M Ozato K Battistini A Critical role of IRF-8 in negative regulation of TLR3 expression by Src homology 2 domain-containing protein tyrosine phosphatase-2 activity in human myeloid dendritic cells J Immunol 2011 186 1951 1962 10.4049/jimmunol.1000918 21220691 PMC4053178

[B36] Salemi J Obregon DF Cobb A Reed S Sadic E Jin J Fernandez F Tan J Giunta B Flipping the switches: CD40 and CD45 modulation of microglial activation states in HIV associated dementia (HAD) Mol Neurodegener 2011 6 3 10.1186/1750-1326-6-3 21223591 PMC3030526

[B37] Masuda T Tsuda M Yoshinaga R Tozaki-Saitoh H Ozato K Tamura T Inoue K IRF8 is a critical transcription factor for transforming microglia into a reactive phenotype Cell Rep 2012 1 334 340 10.1016/j.celrep.2012.02.014 22832225 PMC4158926

[B38] Baur A Garber S Peterlin BM Effects of CD45 on NF-kappa B. Implications for replication of HIV-1 J Immunol 1994 152 976 983 7905504

[B39] Chung KM Liszewski MK Nybakken G Davis AE Townsend RR Fremont DH Atkinson JP Diamond MS West Nile virus nonstructural protein NS1 inhibits complement activation by binding the regulatory protein factor H Proc Natl Acad Sci USA 2006 103 19111 19116 10.1073/pnas.0605668103 17132743 PMC1664712

[B40] Thounaojam MC Kundu K Kaushik DK Swaroop S Mahadevan A Shankar SK Basu A MicroRNA-155 regulates Japanese encephalitis virus induced inflammatory response by targeting src homology 2-containing inositol phosphatase-1 (SHIP1) J Virol 2014 88 4798 4810 10.1128/JVI.02979-13 24522920 PMC3993824

[B41] Ceppi M Pereira PM Dunand-Sauthier I Barras E Reith W Santos MA Pierre P MicroRNA-155 modulates the interleukin-1 signaling pathway in activated human monocyte-derived dendritic cells Proc Natl Acad Sci USA 2009 106 2735 2740 10.1073/pnas.0811073106 19193853 PMC2650335

[B42] Xiao B Liu Z Li BS Tang B Li W Guo G Shi Y Wang F Wu Y Tong WD Guo H Mao XH Zou QM Induction of microRNA-155 during Helicobacter pylori infection and its negative regulatory role in the inflammatory response J Infect Dis 2009 200 916 925 10.1086/605443 19650740

[B43] Xu C Ren G Cao G Chen Q Shou P Zheng C Du L Han X Jiang M Yang Q Lin L Wang G Yu P Zhang X Cao W Brewer G Wang Y Shi Y miR-155 regulates immune modulatory properties of mesenchymal stem cells by targeting TAK1-binding protein 2 J Biol Chem 2013 288 11074 11079 10.1074/jbc.M112.414862 23449975 PMC3630877

[B44] Sullivan RP Fogel LA Leong JW Schneider SE Wong R Romee R Thai TH Sexl V Matkovich SJ Dorn GW 2nd French AR Fehniger TA MicroRNA-155 tunes both the threshold and extent of NK cell activation via targeting of multiple signaling pathways J Immunol 2013 12 5904 5913 24227772 10.4049/jimmunol.1301950 PMC3863634

[B45] Zhou H Huang X Cui H Luo X Tang Y Chen S Wu L Shen N miR-155 and its star-form partner miR-155* cooperatively regulate type I interferon production by human plasmacytoid dendritic cells Blood 2010 116 5885 5894 10.1182/blood-2010-04-280156 20852130

[B46] Lu F Weidmer A Liu CG Volinia S Croce CM Lieberman PM Epstein-Barr virus-induced miR-155 attenuates NF-kappaB signaling and stabilizes latent virus persistence J Virol 2008 82 10436 10443 10.1128/JVI.00752-08 18753206 PMC2573162

[B47] Tang B Xiao B Liu Z Li N Zhu ED Li BS Xie QH Zhuang Y Zou QM Mao XH Identification of MyD88 as a novel target of miR-155, involved in negative regulation of Helicobacter pylori-induced inflammation FEBS Lett 2010 584 1481 1486 10.1016/j.febslet.2010.02.063 20219467

[B48] Ma X Becker Buscaglia LE Barker JR Li Y MicroRNAs in NF-kappaB signaling J Mol Cell Biol 2011 3 159 166 10.1093/jmcb/mjr007 21502305 PMC3104013

